# Normocalcemic primary hyperparathyroidism is not associated with cardiometabolic alterations

**DOI:** 10.1007/s12020-024-04063-0

**Published:** 2024-10-15

**Authors:** Marco Barale, Federica Maiorino, Alessia Pusterla, Federica Fraire, Lorenzo Sauro, Michela Presti, Noemi Sagone, Ezio Ghigo, Emanuela Arvat, Massimo Procopio

**Affiliations:** 1 Division of Oncological Endocrinology; Department of Medical Sciences, University of Turin, Turin, Italy; 2 Division of Endocrinology, Diabetes and Metabolism; Department of Medical Sciences, University of Turin, Turin, Italy

**Keywords:** Normocalcemic primary hyperparathyroidism, Cardiometabolic, Glucose, Blood pressure, Calcium, Parathormone

## Abstract

**Purpose:**

Cardiometabolic disorders are non-classical complications of hypercalcemic primary hyperparathyroidism (HC-PHPT), but whether this risk connotes normocalcemic PHPT (NC-PHPT) remains to be elucidated. We investigated cardiometabolic alterations in both forms of PHPT, looking for their association with indices of disease activity.

**Methods:**

Patients with HC-PHPT (*n* = 17), NC-PHPT (*n* = 17), and controls (*n* = 34) matched for age, sex, and BMI were assessed for glucose, lipid, blood pressure alterations, and history of cardiovascular events to perform a case–control study at an ambulatory referral center for Bone Metabolism Diseases.

**Results:**

NC-PHPT, in comparison to controls, showed similar glucose (mean ± SD, 88 ± 11 vs 95 ± 22 mg/dl), total cholesterol (199 ± 25 vs 207 ± 36 mg/dl), and systolic blood pressure levels (SBP, 132 ± 23 vs 132 ± 19 mmHg), together with a comparable frequency of glucose alterations (6% vs 9%), lipid disorders (41% vs 50%) and hypertension (53% vs 59%, *p* = NS for all comparisons). Conversely, all these abnormalities were more prevalent in HC-PHPT vs controls (*p* < 0.05). When compared to NC-PHPT, HC-PHPT showed higher glucose (113 ± 31 mg/dl), total cholesterol (238 ± 43 mg/dl), and SBP levels (147 ± 15 mmHg) as well as an increased frequency of glucose (41%) and lipid alterations (77%) and a higher number of cardiovascular events (18% vs 0%, *p* < 0.05 for all comparisons). Among indices of PHPT activity, calcium levels displayed a significant correlation with glucose (R = 0.46) and SBP values (R = 0.60, *p* < 0.05).

**Conclusion:**

NC-PHPT is not associated with cardiovascular alterations. The predominant pathogenetic role of hypercalcemia in the development of cardiometabolic disorders could account for the absence of such alterations in NC-PHPT.

## Introduction

Primary hyperparathyroidism (PHPT) is defined as a chronic excess secretion of parathormone (PTH) as a result of parathyroid hyperplasia, adenoma, or carcinoma [1]. Its classic symptomatic form is characterized by elevated serum PTH and calcium levels associated with skeletal and renal complications such as osteoporosis and fractures, chronic renal failure, nephrolithiasis, and/or nephrocalcinosis [2]. In the last decades, the increasing inclusion of calcium and PTH assays in routine exams led to a considerable increase in the frequency of asymptomatic PHPT, which represents today the most common form of this disease [3]. More recently, a new form, i.e., normocalcemic primary hyperparathyroidism (NC-PHPT), has been recognized [4]. NC-PHPT, which is often discovered during evaluation for nephrolithiasis or suspected metabolic bone disease, is characterized by elevated PTH values together with normal serum calcium levels. The exclusion of all secondary causes of PTH elevation, such as vitamin D deficiency, renal insufficiency, hypercalciuria, use of interfering drugs, alterations in phosphate metabolism, low dietary calcium intake or intestinal malabsorption, is necessary to correctly diagnose NC-PHPT [5].

Over the years, new and more subtle clinical manifestations of PHPT have emerged, including an increased incidence of type 2 diabetes mellitus (DM2) and other conditions of impaired glucose tolerance, such as IFG and IGT, a frequent occurrence of dyslipidemia, along with an increased risk of weight gain and hypertension [6–11]. In fact, pre-clinical and clinical studies, both in healthy people and in PHPT, have suggested that PTH and calcium may be involved in glucose and lipid metabolism homeostasis as well as in blood pressure regulation, with higher levels potentially associated with increased cardiovascular risk [12–15]. In addition, in a previous study, we reported that cardiometabolic alterations may also occur independently of the clinical severity of hypercalcemic primary hyperparathyroidism (HC-PHPT) [16]. Consistently, these disorders are now listed as non-classical complications of HC-PHPT, but whether this risk connotes also NC-PHPT remains to be elucidated. In fact, only few studies investigated this issue [17–22] reporting discrepant results and making it difficult to draw firm conclusions. Of note, a major limitation of most of these studies is an arguable diagnosis of NC-PHPT, with some frequent causes of secondary osteoporosis not excluded by these Authors, leading to possible misclassification of their patients.

Our aim was to investigate glucose and lipid disorders as well as blood pressure homeostasis and cardiovascular events in patients with NC-PHPT and HC-PHPT, looking for the association of these cardiometabolic alterations with the indices of PHPT activity.

## Material and methods

Among people referred consecutively to our Bone Metabolism Diseases Centre from January 2022 until December 2023, seventeen patients with normocalcemic primary hyperparathyroidism (NC-PHPT) were recruited and matched for sex, age (±5 years) and BMI (±2.5 kg/m^2^) to seventeen patients with classical hypercalcemic primary hyperparathyroidism (HC-PHPT) and thirty-four controls, in order to perform an observational cross-sectional study. Considering the exploratory nature of this research, we did not perform a power analysis and sample size estimation in designing the study.

Exclusion criteria include multiple endocrine neoplasia, Paget’s disease of bone, familial hypocalciuric hypercalcemia, secondary hyperparathyroidism and other endocrine disorders or drug therapies interfering with glycolipid metabolism, chronic kidney disease with GFR < 60 ml/min.

History of previous cardio- and cerebrovascular events (myocardial infarction and stroke), nephrolithiasis and/or nephrocalcinosis, and clinical fractures was collected. Family history of diabetes mellitus, arterial hypertension, and cardiovascular events, and the frequency of smoking habits were also evaluated. For each patient we collected weight and height, we calculated body mass index and we measured systolic (SBP) and diastolic blood pressure (DBP). The average of three measurements in a seated position after a 5 min rest was considered as the final SBP and DBP. Moreover, we measured waist circumference (WC), performed midway between the inferior margin of the last rib and the crest of the ilium in a horizontal plane.

Calcium and bone metabolism parameters including parathormone, serum total and ionized calcium, phosphorus, 24 h urinary excretion of calcium and phosphorus, 25 OH vitamin D levels as well as total alkaline phosphatase were evaluated. Moreover, glucose and lipid profiles, specifically glucose, insulin, total and HDL cholesterol, triglycerides, uric acid, and creatinine levels were collected. Insulin resistance was detected by using the homeostasis model assessment of insulin resistance (HOMA-IR), which was calculated through the following formula: [fasting insulin (mcUI/ml)] × [fasting glucose (mmol/l)]/22.5: values greater than 2.5 were considered as suggestive of IR.

Diagnosis of glucose disorders (including IFG, IGT, and DM2) and dyslipidemia were based on medical history and according to WHO [23] and NCEP-ATP III criteria [24], while diagnosis of hypertension was made according to The Task Force for the Management of Arterial Hypertension of the European Society of Hypertension and the European Society of Cardiology [25]. Particularly, concerning the diagnosis of LDL-hypercholesterolemia, with LDL-cholesterol calculated using Friedewald’s formula, we considered a fixed cut-off of 2.6 mmol/l (100 mg/dl) as the recommended target for people at intermediate cardiovascular risk [26].

The diagnosis of HC-PHPT was made on the basis of elevated total or ionized calcium levels and inappropriately elevated PTH values, while the diagnosis of NC-PHPT was defined by at least two consecutive determinations, on a 3–6 months period of normal total calcium corrected for albumin and ionized calcium, with persistently elevated PTH levels [4, 5]. The diagnosis of NC-PHPT was confirmed only after the exclusion of all secondary causes of hyperparathyroidism such as renal diseases, vitamin D deficiency, low oral calcium intake, hypercalciuria, calcium malabsorption, alterations in phosphate metabolism or use of interfering medications (e.g., thiazide diuretic or lithium).

All patients underwent upper abdominal ultrasound to exclude renal complications, even asymptomatic, and they were assessed for vertebral fracture by performing vertebral morphometry by DXA.

Serum total calcium (Ca, mmol/l) and phosphate (P, mmol/l) were tested using automated methods based on colorimetric and enzymatic assays (Cobas, Roche). For serum iCa (mmol/l) a specific probe was used after correction for pH. Intact PTH assay (ng/l) based on an immunoradiometric sandwich method (IRMA) that used two polyclonal antibodies (DiaSorin): an antibody, recognizing the C-terminal region (aa 39–84) was used as the capture antibody while an antibody recognizing the N-terminal region was used for detection. Serum 25 OH vitamin D (nmol/l) was tested by a radioimmunoassay method using an antibody with specificity to 25 OH vitamin D (DiaSorin). ALP (UI/l) was tested using colorimetric assay in accordance with a standardized method (Cobas, Roche): in the presence of magnesium and zinc ions, p-nitrophenyl phosphate was cleaved by phosphatases into phosphate and p-nitrophenol, proportional to the ALP activity, that was measured photometrically. Calciuria and phosphaturia were evaluated by colorimetric methods.

Plasma glucose, serum total and HDL cholesterol, triglycerides, and creatinine levels were measured by enzymatic colorimetric tests (Cobas, Roche). Serum insulin was measured by a Solid-phase immunometric assay (Immulite, Siemens) in the subgroup of non-diabetic patients (*n* = 11 among HC-PHPT, *n* = 16 among NC-PHPT, *n* = 30 among controls); the sensitivity of the insulin assay was 2 mU/ml while the intra and the inter-assay coefficients of variation were 4.0 and 4.9%, respectively. Glomerular filtration rate (GFR) was calculated according to Cockroft–Gault formula.

Bone mineral density (BMD) was assessed by dual energy x-ray absorptiometry (DXA) on a Hologic QDR 4500 instrument (Bedford, MA) and expressed as T-score. BMD was evaluated at all three sites (spine, hip, and radius) in patients with PHPT, while lumbar and femoral scans were performed in the controls. Moreover, the following indices were assessed by DXA: 1) the fat mass index (FMI), determined by dividing fat mass (in kilograms) by height squared (in meters), with reference values according to NHANES [27]; 2) the android to gynoid (A/G) ratio, with the android region defined by a caudal limit placed at the top of the iliac crest, with height set to 20% of the distance from the top of the iliac crest to the base of the skull, whereas the gynoid region defined by an upper limit set a distance 1.5 times the height of the android region below the iliac crest, and a lower limit set a distance of 2 times the height of the android region below the iliac crest [28]; an A/G ratio <1 or 1 have been considered as indices of gynoid or android fat distribution, respectively; 3) the fat mass ratio (FMR), defined as the ratio between the percent of the trunk fat mass and the percent of the lower-limb fat mass.

Data are presented as mean ± SD for continuous variables or as percentage for categorical variables. Normality of frequency distribution functions was tested by the Shapiro-Wilk test. Significant differences were sought by the Mann–Whitney U-test or Pearson’s X2 analysis. Spearman’s R coefficient was used to look for associations of PTH and calcium with cardio-metabolic parameters. Calculations were performed using SPSS Windows release 24.0; *p* <0.05 was considered significant.

All procedures performed in the study were in accordance with the ethical standards of the institutional and/or national research committee (Comitato Etico Interaziendale A.O.U. Città della Salute e della Scienza di Torino—A.O. Ordine Mauriziano—A.S.L. Città di Torino) and with the 1964 Helsinki declaration and its later amendments or comparable ethical standards.

## Results

Clinical, biochemical, and demographic data of patients with HC-PHPT, NC-PHPT, and controls are reported in Tables 1 and 2. The frequency of smoking habit as well as the frequency of family history of cardiovascular events, hypertension, and diabetes mellitus were similar between patients with HC-PHPT, NC-PHPT, and controls (data not shown).

**Table 1:** Anthropometric data, body composition indices, and cardio-metabolic parameters in patients with primary hypercalcemic (HC-PHPT) and normocalcemic (NC-PHPT) hyperparathyroidism and in controls (CTRL)

Variables	HC-PHPT (*n* = 17)	NC-PHPT (*n* = 17)	CTRL (*n* = 34)	Reference range
Age (years)	70 ± 9	70 ± 9	70 ± 9	–
Sex (%M)	5 (29)	5 (29)	10 (29)	–
BMI (kg/m^2^)	29 ± 6	28 ± 4	29 ± 5	18.5–24.9
WC (cm)	97 ± 18	91 ± 9	97 ± 12	≤102 M; ≤88 F
FMI (kg/m^2^)	11.0 ± 4.1	11.0 ± 2.1	11.2 ± 4.1	3–6 M; 5–9 F
A/G ratio	1.00 ± 0.16	1.03 ± 0.28	0.97 ± 0.25	<1.00
FMR	1.0 ± 0.17	0.9 ± 0.2	0.9 ± 0.2	<1.26 M; <1.329 F
Glucose (mg/dL)	113 ± 31*#	88 ± 11	95 ± 22	70–99
Insulin (pmol/L)	10.0 ± 6.0	5.6 ± 2.9	11.7 ± 8.4	<30
HOMA-IR	2.7 ± 1.5	1.1 ± 0.5	2.6 ± 2.0	<2.5
Total cholesterol (mg/dL)	238 ± 43*#	199 ± 25	207 ± 36	<200
HDL cholesterol (mg/dL)	64 ± 21	53 ± 14	60 ± 13	>35 M; >40 F
Triglycerides (mg/dL)	145 ± 67	101 ± 39	121 ± 51	<150
LDL Cholesterol (mg/dL)	144 ± 36#	126 ± 27	123 ± 34	<130
SBP (mmHg)	147 ± 15*#	132 ± 23	132 ± 19	90–140
DBP (mmHg)	81 ± 15	76 ± 13	77 ± 12	60–90
Creatinine (mg/dL)	0.78 ± 0.20	0.79 ± 0.19	0.93 ± 0.34	0.5–1.2
GFR (mL/min)	92 ± 21	77 ± 15	72 ± 12	>90
Uric Acid (mg/dL)	5.5 ± 1.1	5.3 ± 0.4	6.1 ± 2.0	3.4–7.0 M; 2.4–5.7 F
Nephrolithiasis or calcinosis	5 (29)#	3 (18)#	0 (0)	–
Morphometric fractures	3 (18)	4 (24)	6 (18)	–

Data are expressed as mean ± standard deviation or as n (%)

*BMI* body mass index, *WC* waist circumference, *FMI* fat mass index, *A/G* android/gynoid ratio, *FMR* fat mass ratio, *HOMA-IR* homeostasis model assessment for insulin resistance, *SBP* systolic blood pressure, *DBP* diastolic blood pressure, *GFR* glomerular filtration rate

*p < 0.05 vs NC-PHPT; #*p* < 0.05 vs CTRL

**Table 2:** Calcium-phosphorus metabolism parameters and densitometric values in patients with primary hypercalcemic (HC-PHPT) and normocalcemic (NC-PHPT) hyperparathyroidism and in controls (CTRL)

Variables	HC-PHPT (*n* = 17)	NC-PHPT (*n* = 17)	CTRL (*n* = 34)	Reference range
PTH (pg/mL)	99 ± 42*#	60 ± 25#	29 ± 21	–
Total calcium (mmol/L)	2.7 ± 0.2*#	2.4 ± 0.2#	2.3 ± 0.2	2.2–2.6
Ionized calcium (mmol/L)	1.4 ± 0.1*#	1.2 ± 0.1#	1.1 ± 0.1	1.1–1.3
24 h urinary calcium (mmol/day)	8.5 ± 4.0*#	4.5 ± 2.7	4.6 ± 4.5	2.5–7.5
Phosphatemia (mmol/L)	0.87 ± 0.11*#	0.96 ± 0.15#	1.19 ± 0.27	0.81–1.45
24 h urinary phosphate (mmol/day)	36.6 ± 19#	24 ± 9	20 ± 11	12.9–32.3
25 OH vitaminD (ng/mL)	21.1 ± 9.9*	38.8 ± 8.4#	23.3 ± 10.6	30–50
Total alkaline phosphatase (UI/L)	104 ± 41*#	73 ± 33	72 ± 17	42–141
T-score at lumbar site	−2.5 ± 1.6	−2.0 ± 1.8	−1.2 ± 2.2	−1/+2
T-score at femoral neck	−1.7 ± 1.0	−2.0 ± 1.0	−0.8 ± 2.6	−1/+2
T-score at total hip	−1.3 ± 1.2	−1.6 ± 1.1	−0.7 ± 1.5	−1/+2
T-score at one-third radius	−2.7 ± 1.3	−2.3 ± 0.8	–	−1/+2

Data expressed as mean ± standard deviation

*PTH* parathyroid hormone

*p < 0.05 vs NC-PHPT; #*p* < 0.05 vs CTRL

As reported in Table 2, when compared to controls, both NC-PHPT and HC-PHPT patients showed the expected significant abnormalities of calcium metabolism parameters, with more pronounced alterations in the latter than in the former group. Moreover, HC-PHPT showed higher levels of PTH, total and ionized calcium, 24 h calciuria, and ALP as well as lower values of 25(OH)vitamin D and serum phosphorus than NC-PHPT patients.

**Fig. 1:**
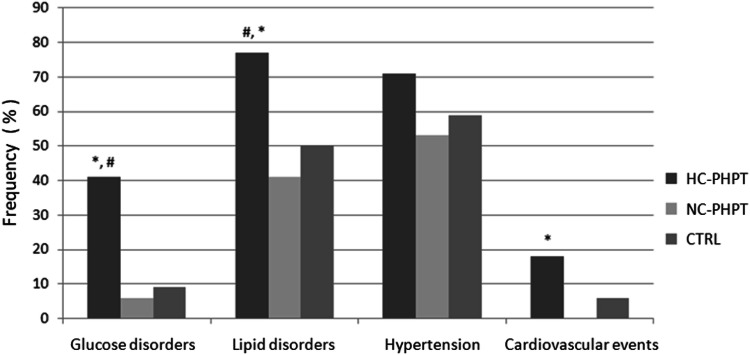
Frequency of glucose and lipid disorders, hypertension, and cardiovascular events in patients with primary hypercalcemic (HC-PHPT) and normocalcemic (NC-PHPT) hyperparathyroidism and in controls (CTRL). *p < 0.05 vs NC-PHPT; #*p* < 0.05 vs CTRL

Cardio-metabolic profile in patients with HC-PHPT, NC-PHPT, and controls is reported in Table 1 and Fig. 1. Concerning glucose metabolism, NC-PHPT showed a similar frequency of glucose disorders (6% vs 9%; *p* = NS), including DM2 (12% vs 12%; *p* = NS), as well as comparable plasma glucose values than controls. Conversely, HC-PHPT in comparison to controls showed a higher frequency of glucose disorders (41% vs 9%), including DM2 (35% vs 12%), as well as increased plasma glucose values (*p* < 0.05 for all comparisons). Accordingly, a higher frequency of glucose disorders and DM2 was found in HC-PHPT than in NC-PHPTC (41% vs 6% and 35% vs 12%), together with higher glucose levels in the former than in the latter group (*p* < 0.05 for all comparisons). Moreover, HC-PHPT were more frequently treated with anti-diabetic drugs than NC-PHPT and controls (41% vs 6% and 6%, respectively).

Serum lipid profile was similar between NC-PHPT and controls with a comparable frequency of lipid disorders. On the contrary, HC-PHPT displayed higher total and LDL cholesterol values as well as a higher frequency of lipid disorders than controls (77% vs 50%; *p* < 0.05). When comparing the two PHPT forms, HC-PHPT showed higher total cholesterol levels and a higher frequency of mixed dyslipidemia (41% vs 12%; *p* < 0.05), hypertriglyceridemia (41% vs 12%; *p* < 0.05) and lipid disorders (77% vs 41%; *p* < 0.05) than NC-PHPT. In addition, patients with HC-PHPT and NC-PHPT were more frequently treated for dyslipidemia (59% and 35%, respectively) than controls (12%; *p* < 0.05).

Blood pressure levels were similar between NC-PHPT and controls, with both groups showing lower systolic blood pressure levels in comparison to HC-PHPT (*p* < 0.05). In addition, the latter group displayed a higher use of anti-hypertensive drugs in comparison to controls (59% vs 29%; *p* < 0.05).

Furthermore, a lower frequency of cardiovascular events was registered in NC-PHPT in comparison to HC-PHPT (0% vs 18% *p* < 0.05).

**Fig. 2:**
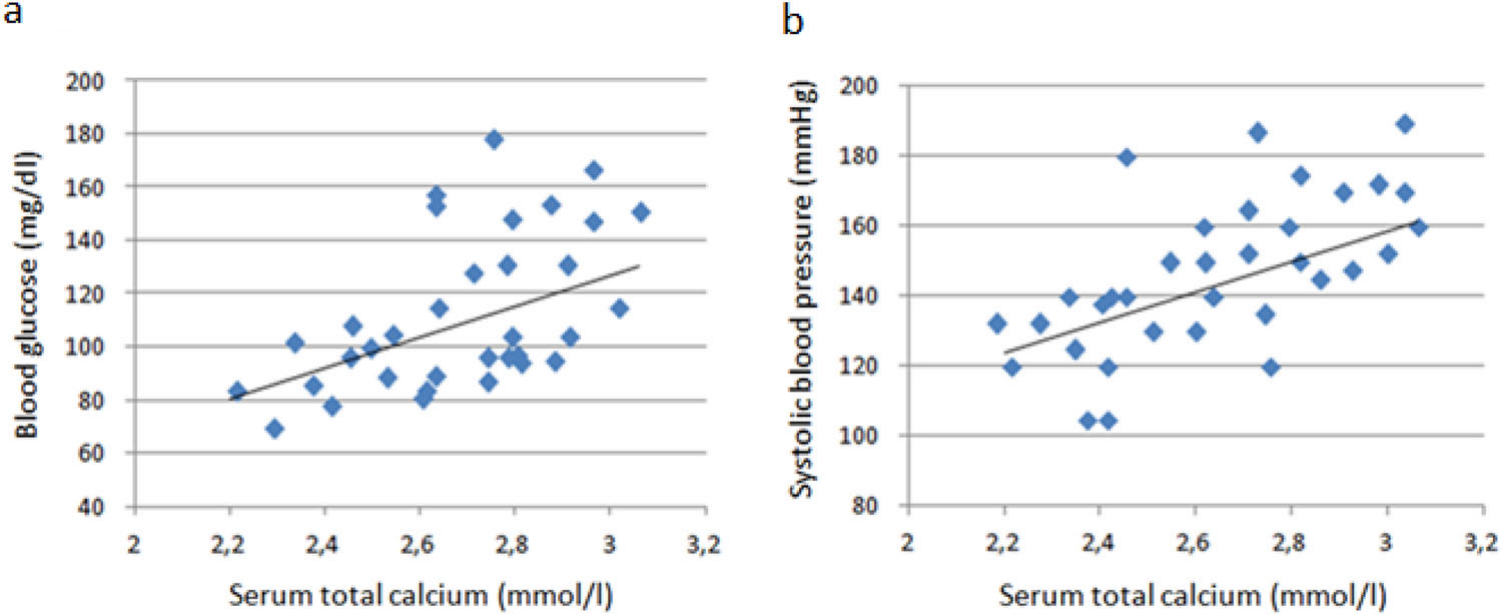
Correlation of serum total calcium with fasting blood glucose (**a**) R = 0.46, *p* < 0.03) and systolic blood pressure. (**b**) R = 0.60; *p* < 0.02) in patients with primary hypercalcemic (HC-PHPT) and normocalcemic (NC-PHPT) hyperparathyroidism

Finally, as shown in Fig. 2, considering all patients with PHPT, we found a positive correlation between total calcium and both glucose (R = 0.46; *p* < 0.05) and systolic blood pressure levels (R = 0.60; *p* < 0.05) together with inverse relationship between serum phosphorus and triglycerides levels (R =− 0.78; *p* < 0.05; data not shown). No significant relationships were found between PTH levels or serum ionized calcium and cardiometabolic parameters. Moreover, considering only HC-PHPT, we found no association of serum calcium and PTH levels with cardiometabolic parameters.

## Discussion

Our findings indicate that NC-PHPT is not associated with cardiometabolic alterations, which conversely have been displayed in our patients with HC-PHPT. Specifically, in our NC-PHPT patients, we found similar glucose, cholesterol, and systolic blood pressure levels, together with a comparable frequency of glucose alterations, lipid disorders, and hypertension in comparison to controls. On the contrary, as expected, HC-PHPT displayed a higher frequency of cardiometabolic disorders compared to controls. In addition, patients with NC-PHPT showed lower glucose, cholesterol, and systolic blood pressure levels as well as a reduced frequency of glucose and lipid alterations and cardiovascular events than HC-PHPT. Among indices of PHPT activity, calcium but not PTH levels displayed a significant correlation with some cardiometabolic parameters.

It is well known that HC-PHPT patients are characterized by a higher prevalence of cardiometabolic disorders, including glucose intolerance, diabetes, dyslipidemia, and hypertension, leading to an increased cardiovascular morbidity and mortality [6–11, 29]. This higher risk connotes HC-PHPT irrespective of its clinical presentation, including asymptomatic patients [16]. On the other hand, few studies investigated the association between cardiometabolic disorders and NC-PHPT, with unclear and controversial results. In line with our findings, Tassone et al. [17] and Cakir et al. [18] reported a similar insulin sensitivity and glucose tolerance between NC-HPHT patients and controls matched for sex, age, and BMI. Conversely, Beysel et al. [19] found higher insulin resistance, increased insulin levels, and HOMA-IR values in the former than in the latter, without any differences between NC-PHPT and HC-PHPT; in addition, in the study by Ozturk et al. [20], NC-PHPT had higher glucose levels than controls despite similar insulin, HOMA-IR, and HbA1c values. Concerning lipid metabolism, Beysel et al. [19] reported higher total cholesterol, LDL cholesterol, and triglycerides levels in NC-PHPT compared to controls, according to previous findings by Hagstrom et al. [21]. However, these results were not confirmed by other studies [18, 20]. In fact, in line with our data, Cakir et al. showed a similar lipid profile between NC-PHPT and controls matched for age, sex, and BMI. In the study by Tordjman et al. [22], NC-PHPT patients displayed similar rates of hypertension when compared to controls, but other previous studies [19, 20] reported higher prevalence of known hypertension and/or increased systolic and diastolic blood pressure levels in the former than in the latter. In our study, despite higher SBP values in HC-PHPT than NC-PHPT and controls, the history of hypertension did not differ between the three groups. Of note, when comparing our results with the few data available from literature, it should be underlined that most of the papers that highlighted a cardiometabolic derangement in NC-PHPT [19–21] were affected by a major bias. Specifically, in the studies by Beysel et al. [19] and Ozturk et al. [20], many patients diagnosed as NC-PHPT presented vitamin D deficiency, whose correction is mandatory to ascertain the disease. Therefore, their patients may not be real NC-PHPT and low vitamin D levels could contribute “per se” to cardiometabolic alterations. Moreover, in the studies by Hagstrom et al. [21], the lack of vitamin D measurement does not allow to exclude that their NC-PHPT patients, histologically proven as PHPT, could be “vitamin D—deficient masked” HC-PHPT, leading to a misclassification. Very little information is available about incidence and prevalence of cardiovascular events in patients with NC-PHPT. In line with our findings, Tordjman et al. reported a higher prevalence of cardio- or cerebrovascular disease in HC- than NC-PHPT patients, despite a similar cardiovascular risk profile between the two groups [22].

Furthermore, we investigated the association of serum calcium and PTH levels with cardiometabolic parameters in HC-PHPT, without obtaining statistically significant results. We ascribed this negative finding to both the lack of a wide distribution of serum calcium and PTH values and the small sample size when considering the groups separately. Thus, to widen the range of serum calcium and PTH levels and to increase the sample size, we chose to perform the same analyses in all PHPT, considering that the statistical validity of the association between the above-mentioned variables is maintained. By including the whole sample of PHPT in our analysis, we found a positive correlation of serum calcium with fasting blood glucose and systolic blood pressure, with PTH levels not significantly related with cardio-metabolic parameters. The predominant role of calcium in the pathogenesis of glucose, lipid, and blood pressure disorders in PHPT is still debated. Previous studies in HC-PHPT have not yet clarified which is the main responsible for the metabolic imbalance and the increase of cardiovascular morbidity and mortality between hypercalcemia and the rise of PTH serum levels. It is well-known that hypercalcemia can compromise glucose tissue uptake by inducing peripheric insulin resistance, then leading to glucose intolerance. Tassone et al., investigating the frequency of glucose intolerance in 122 patients with PHPT in comparison to 61 healthy subjects, found a reduced insulin sensitivity in the former and showed a negative independent association of serum Ca with insulin sensitivity [12]. In addition, hypercalcemia may also have a role in favoring blood pressure disorders in PHPT. Accordingly, Hanson et al. [13] showed that the increase of intracellular calcium in vessel wall cells in PHPT leads to vasoconstriction, with a rise of peripheric resistance and blood pressure. Moreover, in line with our data, Letizia et al. [30] showed an independent positive correlation between ionized calcium and blood pressure in 53 patients with primary hyperparathyroidism. Finally, serum calcium levels have also been correlated to an increase of cardiovascular mortality in PHPT [31], in agreement with our findings showing an increased frequency of cardiovascular events only in HC-PHPT. On the other hand, previous studies suggested that also PTH could be involved in glucose metabolism homeostasis, and blood pressure regulation. Animal studies suggest that PTH underregulate the insulin intracellular signaling mediated by IRS-1, with a consequent increase in peripheric resistance to insulin [32], while Chiu et al. [14] found an inverse correlation of PTH with the insulin sensitivity index (ISI) in a group of 52 healthy subjects. Moreover, it is known that prolonged infusion of parathyroid hormone increases blood pressure probably due to an alteration of blood vessels reactive properties [33] and an increase of blood volume through activation of renin-angiotensin-aldosterone system [34] and a positive correlation of PTH levels with systolic blood pressure has been shown [15].

Based on these findings, one may hypothesize that both high PTH levels and hypercalcemia could contribute to the development of cardiometabolic disorders in PHPT. In this regard, NC-PHPT is an interesting pathological model, that allows us to analyze the contribution of the two factors separately. Our data support a predominant role of hypercalcemia, rather than hyperparathyroidism, in favoring cardiometabolic derangement in PHPT. Nevertheless, it has to be noted that our NC-PHPT patients showed less high PTH levels compared to HC-PHPT. Therefore, it may be that the supposed effect of hyperparathyroidism is weaker in the former than in the latter, according to a low cardiometabolic derangement in NC-PHPT.

Finally, one possible explanation for the lack of association between serum ionized calcium and cardiometabolic parameters is that, despite the theoretically greater accuracy of ionized than total calcium in defining the true biologically active calcium, its precision in clinical practice may be often impaired by many analytical and post-analytical interfering factors.

Our study has some limitations. First, its cross-sectional design did not allow the identification of cause-effect relationships and temporal trends. Second, though considering three study groups with patients well-matched for age, sex, and BMI, the strength of the conclusions is limited by the small sample size, that did not allow us to perform a regression analysis to estimate the effect of exposure adjusted for multiple confounders. Conversely, the major strength of our work is represented by the soundness of the diagnosis of NC-PHPT, according to international guidelines and after the exclusion of all potential causes of secondary hyperparathyroidism.

In conclusion, our data show that cardiovascular alterations do not involve NC-PHPT. The positive correlation of serum calcium levels with fasting blood glucose and systolic blood pressure suggests a predominant pathogenetic role of hypercalcemia rather than hyperparathyroidism in the development of cardiometabolic disorders and could account for the absence of such alterations in NC-PHPT.
